# Recognition in Emergency Department of Septic Patients at Higher Risk of Death: Beware of Patients without Fever

**DOI:** 10.3390/medicina57060612

**Published:** 2021-06-12

**Authors:** Emanuela Sozio, Alessio Bertini, Giacomo Bertolino, Francesco Sbrana, Andrea Ripoli, Fabio Carfagna, Alessandro Giacinta, Bruno Viaggi, Simone Meini, Lorenzo Ghiadoni, Carlo Tascini

**Affiliations:** 1 Infectious Disease Unit, Azienda Sanitaria Universitaria Integrata di Udine (ASU FC), 33100 Udine, Italy; emanuela.sozio@gmail.com (E.S.); alessandro.giacinta@gmail.com (A.G.); c.tascini@gmail.com (C.T.); 2 Department of Medicine (DAME), University of Udine, 33100 Udine, Italy; 3 Emergency Department, North-West District, Tuscany Health Care, Spedali Riuniti Livorno, 57124 Livorno, Italy; alessio.bertini@uslnordovest.toscana.it; 4 Department of Public Health, Clinical and Molecular Medicine, Università degli Studi di Cagliari, 09124 Cagliari, Italy; giacomo.bertolino1985@gmail.com; 5 U.O. Lipoaferesi, Fondazione Toscana “Gabriele Monasterio”, Via Moruzzi,1, 1, 56124 Pisa, Italy; 6 Deep Health Unit, Fondazione Toscana “Gabriele Monasterio”, Via Moruzzi, 1, 56124 Pisa, Italy; ripoli@ftgm.it; 7 SIMNOVA-Interdepartment Centre for Innovative Teaching and Simulation in Medicine and the Health Professions (Centro Interdipartimentale di Didattica Innovativa e di Simulazione in Medicina e Professioni Sanitarie), University of Eastern Piedmont, 28100 Novara, Italy; carfagna.fabio@gmail.com; 8 Neuro Intensive Care Unit, Department of Anesthesia, Careggi Universital Hospital, 50139 Florence, Italy; bruno.viaggi@gmail.com; 9 Internal Medicine Unit, Felice Lotti Hospital, Pontedera, Azienda USL Toscana Nord-Ovest, 1, 56124 Pisa, Italy; simonemeini2@gmail.com; 10 Emergency Medicine, University Hospital of Pisa, 56124 Pisa, Italy; lorenzo.ghiadoni@unipi.it; 11 Department of Clinical and Experimental Medicine, University of Pisa, 56124 Pisa, Italy

**Keywords:** afebrile patients, emergency department, sepsis, septic shock, qSOFA

## Abstract

*Background and Objectives*: Chances of surviving sepsis increase markedly upon prompt diagnosis and treatment. As most sepsis cases initially show-up in the Emergency Department (ED), early recognition of a septic patient has a pivotal role in sepsis management, despite the lack of precise guidelines. The aim of this study was to identify the most accurate predictors of in-hospital mortality outcome in septic patients admitted to the ED. *Materials and Methods*: We compared 651 patients admitted to ED for sepsis (cases) with 363 controls (non-septic patients). A Bayesian mean multivariate logistic regression model was performed in order to identify the most accurate predictors of in-hospital mortality outcomes in septic patients. *Results*: Septic shock and positive qSOFA were identified as risk factors for in-hospital mortality among septic patients admitted to the ED. Hyperthermia was a protective factor for in-hospital mortality. *Conclusions*: Physicians should bear in mind that fever is not a criterium for defining sepsis; according to our results, absence of fever upon presentation might be indicative of greater severity and diagnosis of sepsis should not be delayed.

## 1. Introduction

Sepsis is a life-threatening condition whereby the risk of mortality exceeds that associated with acute coronary syndrome [1,2]. Its definition has been widely debated for several years [3,4], until in 2016 it was defined as “a life-threatening organ dysfunction caused by a dysregulated host response to infection” [5,6,7]. The Sequential Organ Failure Assessment (SOFA) score was proposed as a proxy for evaluating the organ dysfunction occurring during sepsis: an increase by two or more points was established as a necessary diagnostic criterium [5,8].

Notably, the concept of systemic inflammatory response syndrome (SIRS) and its determinants, such as body temperature, are no longer considered.

Sepsis is a time-dependent condition. Hence, early identification and treatment increase chances of survival [9]. As a result, the 2016 Task Force recommended the use of the quick SOFA (qSOFA) score as an early screening tool for discriminating patients with likelihood of sepsis: as qSOFA does not require diagnostic blood testing, it provides an advantage as its timely use may be implemented in every setting [5,10].

The qSOFA score has shown to be a good predictor of mortality, length of hospitalization and requirement of admission in Intensive Care Units (ICU) [11,12,13]. It also proved to be better than the SIRS criteria in identifying septic patients at higher risk of admission in the ICU or death [14,15]. In 2018, a meta-analysis concluded that SIRS criteria are more adequate than qSOFA for the diagnosis of sepsis, while qSOFA is a better predictor of in-hospital mortality [16].

On the other hand, both qSOFA and SIRS criteria are suboptimal predictors of outcome [2], whereas the Early Warning Score (EWS) has demonstrated superiority in selecting the most critically ill among septic patients [17,18].

The incidence of sepsis is increasing worldwide, with an estimated 270 cases per 100,000 inhabitants/year [9,19]. Indeed, most cases initially refer to the Emergency Department (ED) [20]; thus, proper assignment of the priority code at triage could lead to shorter lag time before clinical evaluation and to the administration of the most appropriate treatment [21]. Unfortunately, early recognition of sepsis is still challenging since validated systems and tools for prompt identification are found lacking.

The aim of this study was to define the most accurate mortality outcome predictors for identifying patients with sepsis referring to the ED.

## 2. Materials and Methods

### 2.1. Study Sample and Data Collection

A total of 1014 patients admitted to the ED of Pisa and Leghorn Hospitals, Italy, between March 2017 and December 2019 were included in this retrospective cohort study.

During their stay in the ED, 651 patients had a confirmed diagnosis of sepsis or septic shock (cases) in accordance with the new definitions of sepsis and septic shock (Sepsis-3) [5]. On the other hand, the 363 controls included patients admitted to ED on the same days of the cases, with similar triage diagnosis, which was subsequently corrected with a different condition other than sepsis or septic shock (e.g., consciousness disorders, dyspnea, hypotension, etc.).

Patients meeting sepsis criteria in the ED were identified among patients with infection and a SOFA score of two or more.

Patients who developed septic shock in the ED were identified according to a clinical scheme of sepsis with persisting hypotension requiring vasopressors to maintain MAP ≥65 mmHg and serum lactate levels >2 mmol/L (18 mg/dL) despite adequate volume resuscitation, according to Sepsis-3 definitions [5].

We excluded 114 septic patients whose blood test results or clinical data were not available.

Data were collected from the patients’ records and included the following information: demographic features; risk factors for infection (prosthetic devices, immunosuppression, steroid therapy in the previous 30 days, trauma in the previous 30 days, surgery in the previous 30 days and presence of CVCs and/or bladder catheters); comorbidities (Charlson Comorbidity Index, Cardiovascular disease, Renal insufficiency, Diabetes, COPD, Chronic hepatopathy and Cancer); vital parameters (Body temperature and Mean Arterial Pressure-MAP); clinical parameters for assessing degree of illness (Sequential Organ Failure Assessment-SOFA- Score, quick SOFA-qSOFA, Glasgow Coma Scale-GCS and Shock index), laboratory investigations available in the ED (white blood cells count-WBC-platelet count; bilirubin, creatinine and procalcitonin (PCT) levels; lactate levels in arterial blood); details regarding hospitalization (length of stay and subsequent admission in the ICU); and in-hospital mortality or early death occurring in the ED.

This study did not require an institutional review board oversight due to its retrospective nature and the anonymity of pooled data.

### 2.2. Statistical Analysis

This study aimed primarily at uncovering factors related to in-hospital mortality among the overall septic population of patients referring to the ED. All variables were expressed as mean +/− standard deviation, median and interquartile range or percentage where appropriate. Normality of quantitative variables was assessed with the Shapiro–Wilk test and Q–Q plots. Depending on the distribution of variables, comparisons between groups were performed with unpaired two-tailed *t*-test, Mann–Whitney test or chi-squared test with continuity correction. A *p* value below 0.05 was considered statistically significant. Univariable logistic regression was performed to evaluate the association of each covariate with in-hospital mortality; covariates with a *p* value less than 0.10 were considered for multivariable analysis. A Bayesian averaging of logistic regression multivariable models (BMA) [22] was computed to address model uncertainty, which produces a posterior probability for each possible model and covariate. As a result of BMA, in addition to OR, the probability that the single covariate has a non-zero effect in the final averaged model (posterior probability, *p* (b ≠ 0)) was reported. Covariates with *p* (b ≠ 0) > 0.80 were considered as independently associated to the outcome. Analyses were performed using the R open-source statistical software.

## 3. Results

A total of 1014 patients admitted to the ED were enrolled. Among these, 651 patients received a diagnosis of sepsis or septic shock (cases) while the remaining 363 patients were diagnosed with a different condition other than sepsis or septic shock (controls).

The clinical characteristics of patients are reported in Table 1. The overall median age was 77.7 ± 13.5 years. Most patients were hospitalized (91.3%). Overall, in-hospital mortality was 15.9%.

There were no differences among age and gender between the two patient groups. On the other hand, patients with sepsis required hospitalization more frequently than those within the control group (94.5% vs. 85.7%, <0.0001) and showed higher mortality both early in ED (3.2 vs. 0.56, *p* = 0.0001) and during hospitalization (20.1% vs. 9.7%). Septic shock occurred in 14.8% of sepsis cases admitted to ED but no differences in ICU admissions were observed between the two groups (7.5% vs. 5.2%, *p* = 0.2047).

As opposed to controls, patients with sepsis displayed the following risk factors more frequently: history of trauma within the previous 30 days (6.1% vs. 3.0%, *p* = 0.0428), history of surgery within the previous 30 days (5.2% vs. 0.8%, *p* = 0.0007), presence of central venous catheters—CVCs (8.5% vs. 1.9%, *p* = 0.0001)—and presence of urinary catheters (15.7% vs. 3.3%, <0.0001).

In addition, septic patients were characterized by the following features as opposed to controls: higher body temperature (37.8 ± 1.2 °C vs. 36.9 ± 1.0 °C, *p* < 0.0001); lower MAP (101.0 ± 33.7 vs. 152.8 ± 33.8, *p* < 0.0001), platelet count (/mmc) (200 (142–293) vs. 226.5 (178–291), *p* < 0.0001) and GCS; higher SOFA Score (4 (3–6) vs. 2 (2–4), *p* < 0.00010), shock index (0.9 ± 0.3 vs. 0.7 ± 0.2, *p* = < 0.0001), lactate value (mmol/L) (2.1 (1.2–3.8) vs. 1.2 (0.8–1.9), *p* < 0.0001), white blood cells count (/mmc) (13.4 (9.3–19.6) vs. 10.0 (7.3–13.3), *p* < 0.0001), PCT (ng/mL) (2.9 (0.9–13.1) vs. 0.1 (0.1–0.2), *p* < 0.0001), creatinine (mg/dl) (1.3 (0.9–2.1) vs. 1.0 (0.8–1.4), *p* < 0.0001) and bilirubin (mg/dl) (0.9 (0.6–1.3) vs. 1.0 (0.8–1.4), *p* < 0.0001).

A positive qSOFA was reported in 38.9% of septic patients vs. 8.1% of non-septic controls, *p* < 0.0001.

**Figure 1:**
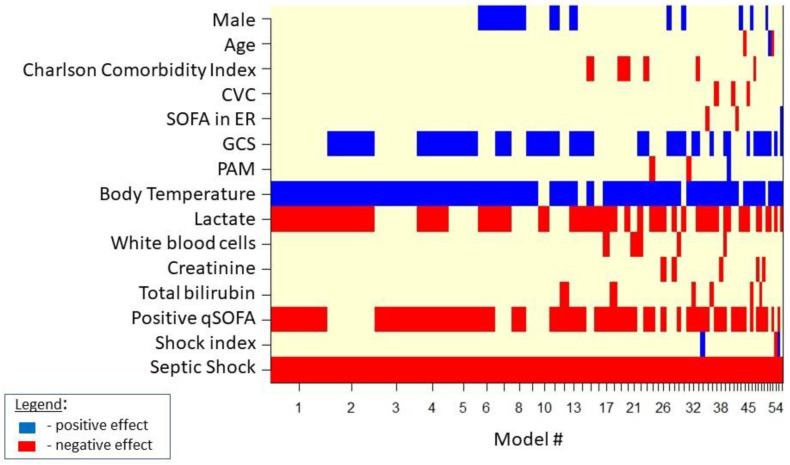
Model selected by BMA for in-hospital mortality in septic cases.

Figure 1 shows the result of the Bayesian model averaging in septic cases. The 50 distinct selected models are indicated on the *x*-axis. In correspondence with each model, the selected variable is marked with a blue rectangle if it is deemed as “protective” (the probability of the event decreases upon its increase). Variables are depicted as red rectangles if selected and deemed as “non-protective” (the probability of the event increases upon its increase). The spacing of the 50 models on the *x*-axis is representative of the posterior probability (of the goodness) of the individual model.

For septic patients admitted to the ED, the Bayesian mean of multivariate logistic regression models (Table 2) identified both septic shock and positive qSOFA as risk factors for in-hospital mortality outcome, while higher temperatures appeared as a protective factor vs. in-hospital mortality outcome.

## 4. Discussion

Sepsis is a time-dependent disease, as reported by a consistent body of evidence [8,23,24]. This implies that early identification and prompt administration of therapy are crucial in order to increase chances of survival. Early recognition of the affected patients is not always easy; indeed, symptoms may be atypical or appear evident only when the condition is very severe [25]. In 2016, along with the new definitions of sepsis and septic shock, both the qSOFA and SOFA scores were proposed as clinical tools for early recognition and definite diagnosis of infections complicated by sepsis, respectively [26].

Despite the uncertainty related to the choice of the best systems of early recognition and hemodynamic management, crucial actions currently recognized for decreasing sepsis related mortality include early recognition and management protocols in the ED [27,28]. When sepsis is readily identified in the ED and severe forms are treated aggressively with sepsis specific care bundles, mortality improvements are significant [8,23,24].

Regarding in-hospital mortality outcomes in septic patients admitted to the ED, our analysis confirmed the presence of septic shock and positive qSOFA as risk factors, while higher body temperature appeared as a protective factor.

Singer et al. demonstrated that qSOFA is a good predictor of mortality, length of hospitalization and ICU admission requirement [11,12,13]. Freund et al. added that qSOFA and SOFA scores are both better than the SIRS criteria at identifying septic patients at risk of death or transfer to the ICU [14,15]. In 2018, a meta-analysis concluded that SIRS criteria are more adequate than qSOFA for diagnosing sepsis, while qSOFA is a better predictor of in-hospital mortality [16].

Our data hereby confirms that qSOFA is a good predictor of mortality in septic patients admitted to ED.

Septic shock is well known to correlate with high mortality rates. Indeed, its definition comprises underlying circulatory and cellular/metabolic abnormalities associated to increased mortality in a subset of patients with sepsis.

The protective role of hyperthermia could have two alternative (but not necessarily mutually exclusive) explanations. Firstly, fever is one of the most prominent symptoms of infection as part of the host acute-phase response to pathogens: It is believed to reduce bacterial growth and promote cytokines synthesis and antibody production, thereby activating immune cell response [29,30]. Secondly, it represents a wake up call that immediately alerts doctors, thus speeding up the diagnostic process [31].

On the other hand, other reports confirm that the presence of hypothermia in patients with severe sepsis was an independent predictor of 28 day mortality and is associated with organ failure [31,32].

A recent study assessed 378 patients admitted to the ED with septic shock. Fever was reported in only 55% of them and afebrile patients had lower rates of antibiotic administration and intravenous fluids. Moreover, the afebrile status was shown to be a significant predictor of in-hospital mortality [33]. Afebrile patients, in our experience, were older and showed higher rates of organ dysfunction.

In recent observations, the absence of fever was associated with suppressed HLA-DR expression over time and findings suggested monocyte dysfunction in sepsis. Afebrile patients had higher rates of 28 day mortality and increased acquisition of secondary infections [34].

Unfortunately, these valuable clinical factors of immune dysfunction have not been taken into account in the present study since they are not commonly used in clinical practice in the ED.

This study has strengths and limitations. Among the study’s strengths, we highlight the use of real-world data, the evaluation of a large number of predictive factors and the availability of the information at admission time in the ED. However, the study has some limitations as well. Firstly, due to the retrospective nature of the study, prospective validations in larger patient cohorts are needed to confirm these preliminary findings. Secondly, retrieving all requested information was at times challenging, as expected in settings burdened by overcrowding such as the ED.

Larger prospective and controlled studies are needed to confirm these findings.

## 5. Conclusions

Early recognition is crucial when managing sepsis. Identifying sepsis is often quite challenging and no single test offers diagnostic certainty in the early stages.

Our data showing hyperthermia as a protective factor for in-hospital mortality suggests the underlying importance of host immune response to sepsis. Furthermore, clinicians should bear in mind that fever is not a criterium for the definition of sepsis. Hence, early diagnosis of sepsis among afebrile patients should not be delayed.

## Figures and Tables

**Table 1:** Comparison between septic patients (*n* 651) and controls (*n* 363).

	Over All (*n* 1014)	Controls (*n* 363)	Sepsis (*n* 651)	*p*
Gender (male)	537 (53.0%)	180 (49.6%)	357 (54.8%)	0.123
Age (years)	77.7 ± 13.5	77.6 ± 13.4	77.8 ± 13.5	0.857
Hospital admission	926 (91.3%)	311 (85.7%)	615 (94.5%)	<0.001
ICU admission	68 (6.7%)	19 (5.2%)	49 (7.5%)	0.205
ED discharge	65 (6.4%)	50 (13.8%)	15 (2.3%)	<0.001
Death in ED	23 (2.3%)	2 (0.56%)	21 (3.2%)	0.012
Death during hospitalization	161 (15.9%)	30 (9.7%)	131 (20.1%)	0.001
Charlson Comorbidity Index	2 (1.0–4.0)	2 (1–4)	2 (1–4)	0.239
Cardiovascular disease	550 (54.2%)	208 (57.3%)	342 (52.5%)	0.163
Renal insufficiency	23 (2.3%)	9 (2.5%)	14 (2.2%)	0.907
Diabetes	250 (24.7%)	105 (28.9%)	145 (22.3%)	0.023
COPD	176 (17.4%)	104 (28.7%)	72 (11.1%)	<0.001
Prosthetic device	157 (15.5%)	73 (20.1%)	84 (12.9%)	0.003
Chronic hepatopathy	46 (4.5%)	18 (5.0%)	28 (4.3%)	0.745
Immunosuppression	88 (8.7%)	33 (9.1%)	55 (8.5%)	0.817
Cancer	145 (14.3%)	66 (18.2%)	79 (12.1%)	0.011
Steroid therapy (in 30 days)	154 (15.2%)	77 (21.2%)	77 (11.8%)	<0.001
Trauma (in 30 days)	51 (5.0%)	11 (3.0%)	40 (6.1%)	0.043
Surgery (in 30 days)	37 (3.7%)	3 (0.8%)	34 (5.2%)	0.001
Presence of CVC	62 (6.1%)	7 (1.9%)	55 (8.5%)	<0.001
Presence of urinary catheter	112 (11.1%)	12 (3.3%)	100 (15.7%)	<0.001
Body temperature (°C)	37.6 ± 1.2	36.9 ± 1.0	37.8 ± 1.2	<0.001
MAP	119.2 ± 41.8	152.8 ± 33.8	101.0 ± 33.7	<0.001
GCS	13.7 ± 3.0	14.2 ± 2.5	13.3 ± 3.2	<0.001
Septic shock	96 (9.5%)	0 (0.0%)	96 (14.8%)	<0.001
Shock index	0.8 ± 0.3	0.7 ± 0.2	0.9 ± 0.3	<0.001
Positive qSOFA	277 (27.3%)	24 (8.1%)	253 (38.9%)	<0.001
SOFA score	3 (2.0–5.0)	2 (2–4)	4 (3–6)	<0.001
Lactate (mmol/L)	1.6 (1.0–2.9)	1.2 (0.8–1.9)	2.1 (1.2–3.8)	<0.001
WBC (/mmc)	11.9 (8.4–16.9)	10.0 (7.3–13.3)	13.4 (9.3–19.6)	<0.001
PCT (ng/mL)	1.4 (0.3–7.8)	0.1 (0.1–0.2)	2.9 (0.9–13.1)	<0.001
Creatinine (mg/dl)	1.2 (0.8–1.8)	1.0 (0.8–1.4)	1.3 (0.9–2.1)	<0.001
Bilirubin (mg/dl)	0.8 (0.5–1.2)	0.5 (0.3–0.9)	0.9 (0.6–1.3)	<0.001
Platelet count (/mmc)	213 (153–293)	226.5 (178–291)	200 (142–293)	<0.001

**Table 2:** Bayesian mean of multivariate logistic regression performed to investigate the association with the in-hospital mortality outcome in septic cases.

Variable	OR	95% CI OR	*p* (B! = 0)
Male	0.902	0.874–0.931	17
Age	1.000	0.999–1.001	1.8
Charlson Comorbidity Index	1.008	1.004–1.013	6.9
CVC	1.009	0.997–1.021	2.3
SOFA score	1.001	0.999–1.002	2.2
GCS	0.936	0.928–0.944	51.6
PAM	1.000	0.999–1.000	2.8
Body Temperature	0.687	0.673–0.701	92.5
Lactate	1.107	1.095–1.120	63.7
White blood cells	1.001	1.000–1.002	5.7
Creatinine	1.004	1.001–1.007	4.1
Total bilirubin	1.011	1.004–1.017	6.4
Positive qSOFA	2.144	2.002–2.297	71.7
Shock index	0.995	0.985–1.005	2
Septic Shock	6.582	6.289–6.88	100

Legend: the variables in italics are those that possess independent effect on mortality. Septic shock and positive qSOFA are risk factors for in-hospital mortality, while higher temperature is a protective factor.

## Data Availability

Data are available upon contacting the corresponding author.
